# Inactivation of a Mismatch-Repair System Diversifies Genotypic Landscape of *Escherichia coli* During Adaptive Laboratory Evolution

**DOI:** 10.3389/fmicb.2019.01845

**Published:** 2019-08-16

**Authors:** Minjeong Kang, Kangsan Kim, Donghui Choe, Suhyung Cho, Sun Chang Kim, Bernhard Palsson, Byung-Kwan Cho

**Affiliations:** ^1^Department of Biological Sciences, Korea Advanced Institute of Science and Technology, Daejeon, South Korea; ^2^KAIST Institute for the BioCentury, Korea Advanced Institute of Science and Technology, Daejeon, South Korea; ^3^Intelligent Synthetic Biology Center, Daejeon, South Korea; ^4^Department of Bioengineering, University of California, San Diego, San Diego, CA, United States; ^5^Department of Pediatrics, University of California, San Diego, San Diego, CA, United States

**Keywords:** adaptive laboratory evolution, mismatch repair, *Escherichia coli*, genotype space, phenotype microarray

## Abstract

Adaptive laboratory evolution (ALE) is used to find causal mutations that underlie improved strain performance under the applied selection pressure. ALE studies have revealed that mutator populations tend to outcompete their non-mutator counterparts following the evolutionary trajectory. Among them, *mutS-*inactivated mutator cells, characterize d by a dysfunctional methyl-mismatch repair system, are frequently found in ALE experiments. Here, we examined *mutS* inactivation as an approach to facilitate ALE of *Escherichia coli.* The wild-type *E. coli* MG1655 and *mutS* knock-out derivative (Δ*mutS*) were evolved in parallel for 800 generations on lactate or glycerol minimal media in a serial-transfer experiment. Whole-genome re-sequencing of each lineage at 100-generation intervals revealed that (1) mutations emerge rapidly in the Δ*mutS* compared to in the wild-type strain; (2) mutations were more than fourfold higher in the Δ*mutS* strain at the end-point populations compared to the wild-type strain; and (3) a significant number of random mutations accumulated in the Δ*mutS* strains. We then measured the fitness of the end-point populations on an array of non-adaptive carbon sources. Interestingly, collateral fitness increases on non-adaptive carbon sources were more pronounced in the Δ*mutS* strains than the parental strain. Fitness measurement of single mutants revealed that the collateral fitness increase seen in the mutator lineages can be attributed to a pool of random mutations. Together, this study demonstrates that short-term mutator ALE extensively expands possible genotype space, resulting in versatile bacteria with elevated fitness levels across various carbon sources.

## Introduction

Bacterial cells are under constant selection pressure to adapt to ever changing environmental conditions. The adaptive response to changing conditions is based on finding fitness-enhancing genetic changes. Adaptive laboratory evolution (ALE) experiments using microbes have identified such mutations and horizontal gene transfer as the key determinants underpinning the emergence of adaptive traits such as antibiotic resistance (Dragosits and Mattanovich, 2013). While adaptive evolution results in the rewiring of cellular regulatory and metabolic networks, mutation rate itself may also be subject to adaptation through a selection pressure on the genes associated with DNA repair (Rosche and Foster, 1999; Giraud et al., 2001b; Sprouffske et al., 2018).

It has been well documented that the emergence of mutator lineages during adaptive evolution results in a more rapid adaptation compared to wild-type strains (Sniegowski et al., 1997; Giraud et al., 2001a; Notley-McRobb et al., 2002; Barrick et al., 2009; LaCroix et al., 2015; Shibai et al., 2017). *In silico* prediction of mutation rate dynamics based on the canonical *Escherichia coli* mutation rates revealed that a 10–100-fold increase in mutation rates facilitates rapid adaptation in the bacterial population (Taddei et al., 1997). Likewise, it has been empirically shown that mutator strains with an intermediary range of mutability harnessed the fittest lineages following adaptation in minimal and rich media alike, and that too high a mutation rate adversely affects successful adaptation (Loh et al., 2010; Ishizawa et al., 2015; Sprouffske et al., 2018), and in some instances, survival (Shibai et al., 2017). Interestingly, mutators generate a plethora of random mutations many of which exert no imminent effect on host fitness. However, mounting evidence suggests the role of non-adaptive mutations in shaping novel adaptive phenotypes in the process of adaptation (Wagner, 2008; Szappanos et al., 2016).

In a prokaryotic population with basal mutation rate, most randomly arising mutations are subjected to loss due to genetic drift. However, in the presence of mutators, selection pressure further promotes the accumulation of intermediary mutations, expanding the pool of genetic reservoir that may serve as secondary adaptive mutations (Lynch and Abegg, 2010). This means that random mutations that are now neutral or slightly deleterious may confer a selective advantage in alternative environments. Previously, it was demonstrated in *E. coli* that random mutations accrued through adaptive evolution on one carbon (propylene glycol) enabled utilization of structurally similar carbon (ethylene glycol) that is inaccessible to wild-type *E. coli* K-12 (Szappanos et al., 2016). Another study showed that moderate mutator strains provide a collateral, other than adaptive, survival advantages in the presence of extreme stressors including direct exposure to antibiotics, toxic heavy metals and chemicals (Sprouffske et al., 2018).

We thus sought to investigate whether ALE of mutator cells can be leveraged to expand the fitness landscape of *E. coli* across an array of non-adaptive environments. To this end, we engineered a methyl-mismatch repair (MMR)-inactivated (Δ*mutS*) mutator *E. coli* strain with 50- to 800-fold increase in mutation rates (LeClerc et al., 1996; Rosche and Foster, 1999; Zhu et al., 2014; Ishizawa et al., 2015). The MMR system is a highly conserved post-replicative repair machinery found across prokaryotes to eukaryotes. This system governs genomic stability through correction of base-pair mismatches, small insertions and deletions on nascent daughter DNA strands, and by preventing recombination between homologous DNA segments (Modrich, 1991). In *E. coli*, the MMR system is comprised of three proteins with mutually exclusive functions – MutHLS. Among them, MutS protein initiates the MMR activity through strand-specific mismatch recognition and simultaneous activation of cognate MMR proteins. Defects in MutS function hence compromises the repair activity of MMR system, rendering the host cell more potent toward mutation (Modrich, 1991; Tsuru et al., 2015). The Δ*mutS* mutator and wild-type *E. coli* K-12 MG1655 strains were serially propagated on two different carbon-limiting conditions (glycerol and lactate) for 800 generations. To elucidate the genetic basis of ALE, we analyzed the mutation profiles by whole-genome re-sequencing at 100-generation intervals. We also assayed the fitness profiles of the evolved Δ*mutS* mutator strain, the parental strain, and the wild-type counterparts in both adaptive and non-adaptive environments. Together, our results suggested that moderate mutator strains, such as *mutS*-inactivation, confer a higher-on-average fitness advantage on alternative, non-adaptive environments.

## Materials and Methods

### Strain Construction and Growth Conditions

All strains used are *E. coli* K-12 MG1655 and its derivatives (Supplementary Table S1). The *mutS* mutator strain (Δ*mutS*) was constructed by a one-step inactivation method as described previously (Datsenko and Wanner, 2000). Homologous lambda recombination was used to obtain single nucleotide modification in the wild-type and Δ*mutS* mutator strain genomes. The mutant and the wild-type gene constructs were amplified from the genomic DNA of either wild-type or the end-point populations carrying the variant of interest with appropriate primers (Supplementary Table S2). A second PCR was performed using plasmid pKD13 as the template using proper primers (Supplementary Table S2). The two PCR amplicons were fused into gene-FRT-neo^R^-FRT by overlap extension PCR (Urban et al., 1997). Downstream procedures including λ-Red recombinase expression, FLP-FRT-mediated marker elimination and single clone isolation were performed by the same protocol for a one-step inactivation method (Datsenko and Wanner, 2000). Lastly, the presence of the desired variants – *add* (947C > T), *xdhB* (860insG), and *ilvH* (124A > G) – and the corresponding wild-type counterparts were confirmed using Sanger sequencing.

For ALE experiment, cells were cultured in M9 minimal medium supplemented with 2 mM of MgSO_4_, 0.1 mM of CaCl_2_, and either 0.2% (w/v) of lactate or 0.2% (v/v) glycerol as the sole carbon source. Cells were incubated at 30°C unless stated otherwise. For growth rate measurement on non-adaptive carbon/nitrogen sources, M9 minimal medium containing 0.2% 2′-Deoxyadenosine (w/v) + 0.2% lactate (v/v), 0.2% inosine (w/v) + 0.2% glycerol, 0.2% L-Threonine + 0.2% lactate and 0.2% pyruvate (w/v) were used. For fitness assay, cells were pre-cultured in M9 minimal medium containing appropriate carbon or nitrogen sources then serially passaged for two times (Shachrai et al., 2010). To quantitatively determine cellular growth kinetics, cell density was measured on EPOCH2 plate readers (BioTek, Winnoski, VT, United States) and Synergy H1 (BioTek, Winnoski, VT, United States) at 30°C with shaking (807 rpm double orbital) for 24 h, using default 48-well configuration. Absorbance at OD_600 nm_ was measured at 10-min intervals. The resulting data output was analyzed on the Gen5 software.

### Adaptive Laboratory Evolution

A total of eight independent lineages comprising two biological replicates from each strain in either M9 lactate or M9 glycerol media. All lineages were adaptively evolved for ∼800 generations. An arbitrarily chosen single colony on LB agar plate was inoculated on 3 mL LB medium then incubated overnight; this batch served as the ancestral stock cells and was kept frozen in −80°C, 20% (v/v) glycerol. Simultaneously, 100 μL of the overnight culture was serially transferred to 3 mL of either 0.2% glycerol or lactate M9 minimal media under the same culture conditions; these cultures were used to seed the main ALE culture at an initial OD_600 nm_ of ∼0.02 in 60 mL of M9 media in 300 mL Erlenmeyer flask. The cells were allowed to propagate until the mid-exponential growth phase (OD_600 nm_ = 0.4–0.6), then serially passaged to fresh M9 media up until 800 accumulative generations were reached. The passaging was accompanied with an intermittent decrease in initial OD_600 nm_ to compensate for the adaptive increase in growth rates. Cell pellets from every passage were collected using a centrifuge and suspended in 20% glycerol (v/v) for − 80°C storage. Contamination screening was performed in several ways. Cross-contamination between wild-type and Δ*mutS* mutator strain was checked by PCR with primer sets that hybridize to a region flanking *mutS* gene (Supplementary Table S2). PCR reaction was set up total 20 μL reaction volume, comprised of 10 μL of HotStarTaq Master Mix (Qiagen, Hilden, Germany), 0.4 μL of forward and reverse primers, and 100 ng of genomic DNA. All PCR reactions were conducted in the C1000^TM^ Thermal Cycler (Bio-Rad Laboratories, Hercules, CA, United States). A single band of ∼2,900 bp corresponds to the intact *mutS* gene of MG1655 wild-type, while a 300 bp-sized band indicates the *mutS-* deletion in Δ*mutS* mutator strain.

### Fitness Measurements

We estimated the doubling time of our evolving lineages at every passage as a proxy for their fitness. Generation per passage was calculated from Eq. 1, where OD*_*i,n*_* and OD*_*f,n*_* are initial and final OD_600 nm_ of *n*th passage, respectively. Accumulated generation is the sum of all generations elapsed (Eq. 2) where *f* is the final passage. Doubling time per passage was estimated using Eq. 3. The first passage was intentionally excluded to dilute physiological constraints ensuing from stationary phase growth rate in preceding cultures (Shachrai et al., 2010).

(1) $$\ln\left(\frac{{\text{OD}}_{f,n}}{{\text{OD}}_{i,n}}\right)/\ln2$$

(2) $$\sum_{n=1}^{f}\frac{\ln\left(\frac{{\text{OD}}_{f,n}}{{\text{OD}}_{i,n}}\right)}{\ln2}$$

(3) $$\frac{\mathrm{time}\,\mathrm{per}\,\mathrm{passage}\,\left(h\right)}{\mathrm{generation}\,\mathrm{per}\,\mathrm{passage}}$$

### Competition ALE

MG1655 wild-type and Δ*mutS* mutator strain were inoculated in 1:1 ratio in cell density (OD_600 nm_) on 60 mL of 0.2% glycerol and lactate M9 minimal media in duplicate and were serially cultured as per the ALE method described above. The sole genetic marker was *mutS* gene to differentiate between the two strains. Strain profiles in mix-culture were periodically confirmed using colony PCR with two primer sets – a universal primer set hybridized to both wild-type and *mutS*^–^genome, and a “*mutS*+” primer set specific to *mutS* gene that discriminates MG1655 wild-type from *mutS*^–^ genome (Supplementary Table S2). Each PCR reaction was set up total 20 μL reaction volume, comprised of 10 μL of Accupower Master Mix (Bioneer, Daejeon, South Korea), 0.4 μL of forward and reverse primers, and 0.5 μL of ALE culture. Serial passaging was performed up until one of the two strains was eliminated from the competition ALE as indicated by the absence of corresponding amplicon band(s) from the PCR reaction.

The ratio of the competing populations over time was quantified using real-time qPCR. Since the amount of genomic DNA is proportional to cell density, by determining the relative quantity of genomic DNA from the mix-culture, the ratio of heterogeneous populations present at specific time points was derived. As *mutS* deletion was the sole identifier of Δ*mutS* mutator strain, we designed two primer sets, one targeting *mutS* gene and the other one flanking the scar region on the *mutS*^–^ genome (Supplementary Table S2), yielding amplicons of 108 and 106 bp, respectively. To demonstrate the feasibility of this experiment, wild-type and Δ*mutS* mutator strain were mixed in the ratios of 1:9, 3:7, 5:5, 7:3, and 9:1 based on the optical density at 600 nm. Each qPCR reaction was wet up total 20 μL reaction volume, composed of 10 μL KAPA SYBR^®^ FAST qPCR Master Mix (2×) Universal (KAPA Biosystems, Wilmington, MA, United States), 0.4 μL of 10 mM forward and reverse primers, and 1.0 μL of 1 mM template DNA. A 2-step thermal cycle program was performed according to the following program: –95°C for 3 min, –95°C for 3 s, –66°C for 20 s, repeat the last two steps for 30 cycles, and 12°C for holding. Relative fluorescence unit (RFU) was detected using the CFX96 Touch^TM^ Real-Time PCR Detection System (Bio-Rad Laboratories, Hercules, CA, United States). The empirical ratio between wild-type and Δ*mutS* mutator populations was computed from 1/(2^∧^(Cq_B_ – Cq_A_)), where Cq_B_ and Cq_A_ are quantification cycles from primer B and A. Linear regression between theoretical and empirical ratio yielded *R*^2^ values of 0.99, demonstrating that direct quantification of gDNA can be used to infer mixed population ratios. The strain ratio is inferred by comparing the threshold cycle (C_T_) to the standard curve. All samples were analyzed in duplicates.

### Whole Genome Resequencing

A total of 66 samples were sequenced including (i) 64 population samples at 100 generation intervals from the eight independently evolved lineages and, (ii) two parental strains of *E. coli* MG1655 wild-type and Δ*mutS* mutator strain. Genomic DNA (gDNA) was isolated using a Wizard Genomic DNA Purification Kit (Promega, Madison, WI, United States) as per the manufacturer’s protocol. The isolated gDNA was checked using a NanoDrop^TM^ 2000 (ThermoFisher Scientific, Waltham, MA, United States) to assure UV absorbance ratio between 1.8 ∼ 2.0 and visualized under 1% agarose gel. The DNA concentration was quantified via a Qubit^®^ 2.0 Fluorometer (Invitrogen, Carlsbad, CA, United States) using a Qubit^TM^ dsDNA HS Assay Kit (Invitrogen, Carlsbad, CA, United States). For each sample, 2,200 ng input DNA was suspended in 56 μL of TE Buffer (pH 8.0) and sheared to target peak size of 400–500 bp using the Covaris S220 Focused-ultrasonicator (Covaris, Woburn, MA, United States) according to manufacturer’s recommendations, except that sonication duration was subjected to variation. NGS library was prepared using a TruSeq DNA PCR-Free kit (Illumina FC-121-3002) following the manufacturer’s guide. Resulting libraries were sequenced with rapid-run mode as 50 cycle single-ended reaction in the Illumina Hiseq 2500 system.

### Mutation Profiling

Sequencing data were processed on a CLC Genomics Workbench (CLC bio, Aarhus, Denmark). Adapter sequences were trimmed using a Trim Seuqnec Tool in NGS Core Tools with a quality limit of 0.05. Reads containing more than two ambiguous bases were omitted and the resulting reads were mapped to the *E. coli* MG1655 reference genome (NCBI accession NC_000913.3) with following parameters: mismatch cost: 2, indel cost: 3, length and similarity fractions: 0.9. Variant calling was performed using a Quality-based Variant Detection Tool with the following parameters: neighborhood radius: 5, maximum gap and mismatch counts: 5, minimum neighborhood and central qualities: 30, minimum coverage: 10, minimum variant frequency: 10%, and maximum expected alleles: 4. Non-specific matches were ignored, and bacterial and plant plastid genetic codes were used. Variants in non-specific regions and repeat regions (i.e., rRNA and transposases) were discarded. The variants from the biological replicates were merged together to generate an exhaustive view of the genotypic landscaped shaped in the course of adaptive evolution. We considered gene, rather than gene position, as a unit for mutation. As for the mutations that overlap between the biological replicates, mutations with higher frequency was chosen.

### Phenotype Microarray

We comparatively analyzed the growth capacity of *E. coli* MG1655 and the evolved strains on non-evolutionary carbon sources in Biolog PM1 MicroPlate^TM^ (Biolog Inc., Hayward, CA, United States), which contains 95 different carbon sources as a sole nutrient source. The frozen cell stocks were inoculated in 3 mL of 0.2% lactate or glycerol M9 minimal media, cultured overnight at 37°C with agitation. Two technical replicates were tested per sample. Cells were collected using a centrifuge, the cell pellets re-suspended in 6 mL of Inoculating Fluid A (Biolog Inc., Hayward, CA, United States) with OD_600 nm_ of approximately 0.4 (42% T cell). To make 85% T cell suspension, 4 mL of 42% T cell suspension was transferred to 20 mL of Inoculating Fluid A. 100 μL of 85% T cell suspension was loaded onto each well on the PM01 plate. To quantitatively determine cellular growth kinetics, cell density was measured on EPOCH 2 plate readers (BioTek, Winnoski, VT) at 30°C with shaking (807 rpm double orbital) for 24 h, using default 96-well configuration.

### Analysis of PM01 Data

Doubling time measured from the negative-control sample (well A01 of the Biolog PM01 plate) grown in nutrient-free Inoculating Fluid A was used as the threshold for growth rate. Substrates with doubling time equal or less than the threshold value were designated as “no growth rate” and were excluded for further analysis. Specific growth rate of each substrate was estimated using the BioTek Gen5 software. Growth rate improvement or retardation of the end-point strains relative to the ancestral strains was calculated as log_2_ (fold-change), and statistical significance was computed between the technical replicates using the student’s independent *t*-test (normal distribution was assumed), and logarithmically expressed in –log_10_ (*P*-value). The substrates with statistically non-significant fitness changes (*P* > 0.05, Student’s independent *t*-test) were omitted from subsequent analysis. Those with significant changes were categorized into four groups: (i) Gain of Function – refers to substrates on which the adapted strains acquired the ability to grow; (ii) Loss of Function – instance where the cell growth becomes diminished in the adapted strains; (iii) Fitness gain– denoting substrates on which significant enhancement in the growth capacity is seen following adaptive evolution; and (iv) Fitness loss – substrates with significant retardation in growth rate following adaptive evolution.

## Results

### Adaptive Laboratory Evolution in Lactate or Glycerol Minimal Media

**FIGURE 1:**
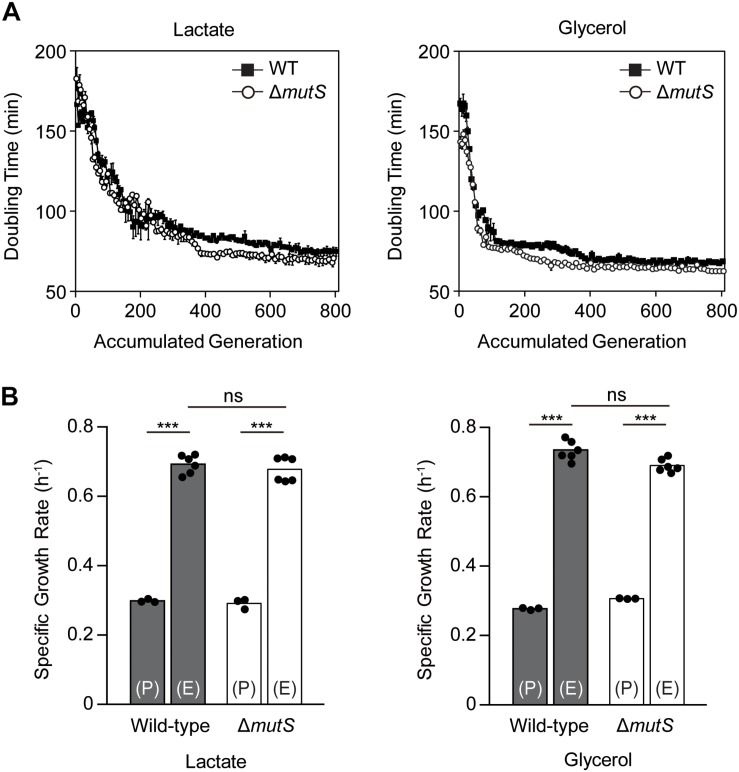
An overview of the fitness trajectory of *E. coli* MG1655 wild-type and Δ*mutS* mutator strains adaptively evolved in M9 lactate or glycerol minimal media over 800 generations. **(A)** Estimated doubling time as the function of accumulated generation. Filled and blank points represent the wild-type and the Δ*mutS* strains respectively. Error bars indicate SD. **(B)** Specific growth rate of the ancestral and the end-point ALE populations of the wild-type and the Δ*mutS* strains. The black dots indicate the data points in place of error bars. Key: (P) – wild-type lineage; (E) – mutator lineage; ns, *P*-value > 0.05; ^∗∗∗^*P*-value < 0.001.

Two biological replicates from each of *E. coli* K-12 MG1655 wild-type and *mutS* knock-out derivate (Δ*mutS* mutator) strains were propagated in M9 minimal medium containing either lactate or glycerol. ALE yielded two lactate-adapted wild-types, two lactate-adapted mutators, two glycerol-adapted wild-types and two glycerol-adapted mutators evolved for 800 generations using serial transfer. To characterize the fitness trajectories accompanying the ALE, doubling time per passage was estimated as a function of accumulated generations (Wiser and Lenski, 2015). The fitness trajectories revealed that the ALE was the most rapid in the first 200 generations (Figure 1A). By the 800th generation, fitness increase reached a plateau in all ALE lineages. Although the fitness trajectories were altogether similar between the two strains, the early trajectories showed that the doubling time of Δ*mutS* mutator strain decreased faster than that of the wild-type strain. Specific growth rates of the end-point populations in each lineage were then calculated to assess the outcome of mutator-driven evolution on adaptive fitness (Figure 1B). Specific growth rate of the Δ*mutS* end-point population was 2.45 ± 0.17 fold higher than that of its parental strain, which was only marginally higher than that of the wild-type lineage (2.29 ± 0.09 fold increase). Taken together, despite of some differences in the rate of fitness gain in the course of ALE, the final fitness levels attained by the end-point populations of the wild-type and the Δ*mutS* mutator strains were comparable.

### Mutator Strain Outcompetes Its Wild-Type Counterpart in Competition Experiments

**FIGURE 2:**
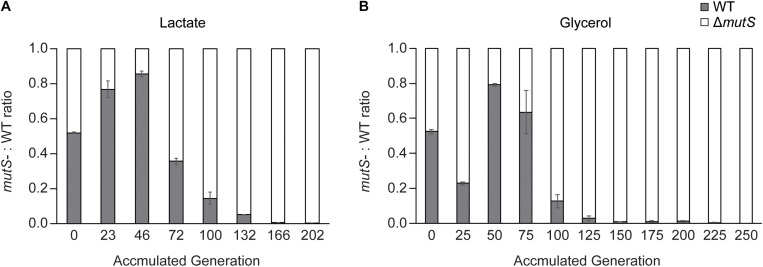
Competition experiment between *E. coli* parental wild-type and Δ*mutS* strains. Ratio-wise representation of the proportion of the parental wild-type versus the parental Δ*mutS* strains in the course of competition experiment in **(A)** lactate or **(B)** glycerol minimal medium. Each bar represents the Δ*mutS*/wild-type ratios from the propagating cultures sampled intermittently.

To recapitulate the observation that increased mutagenic potential confers a selective advantage on a short-term adaptation (Chao and Cox, 1983; Giraud et al., 2001a), we performed competitive growth experiments between the wild-type and the Δ*mutS* mutator strains and evaluated the relative fitness levels of the two strains. The two strains were mixed in 1:1 ratio on M9 lactate or glycerol minimal media respectively, at starting OD_600 nm_ of 0.05 in duplicate. In these growth conditions, the strains share limited resources and compete for survival until a dominant strain takes over the population during a short-term competition. In all four mixed cultures, the Δ*mutS* mutator strains eventually outcompeted their DNA repair-proficient counterparts (Figure 2A). In each of lactate or glycerol serial culture, Δ*mutS* mutator strains completely displaced the wild-type strains (<0.1%) approximately by the 200th generations, indicating that acquisition of beneficial mutations was faster in the Δ*mutS* mutator strains than in the wild-type strains.

Until the 46th generation of the lactate competition experiment, the wild-type strains constituted up to 80% of ∼ 4 × 10^4^ cellular population largely owing to its faster initial growth rate (Figure 2A). By the 72nd generation, the Δ*mutS* mutator strains occupied more than half of the population, then gradually displaced the wild-type counterpart down to < 0.1% by the 202nd generation (Figure 2A). The fluctuation on previous strain-independent ALE experiment results (Figure 1) coincided with these results, where the wild-type strains showed superior growth rate than the Δ*mutS* mutator strain on M9 lactate at the starting point, it became reversed by the 50th generations. Meanwhile, until the 25th generation of the glycerol competition experiment, the Δ*mutS* mutator strains occupied about three quarter of the population. This was expected as the specific growth rate of the ancestral Δ*mutS* mutator strains was faster than the ancestral wild-type strains on M9 glycerol (Figure 1B). Yet, this was immediately followed by a marked increase in the wild-type population making up nearly 80% of the total population. Such rapid fitness increase in the wild-type strains was also evident in the individual ALE experiments, where the growth rate of the wild-type strains evened with that of the Δ*mutS* mutator strains by the 40th generation on M9 glycerol media condition (Figure 1A). In subsequent generations, the Δ*mutS* mutator strains serially displaced the wild-type counterparts down to 0.1% by the end of the short-term laboratory evolution experiments (Figure 2A). These results confirm that the mutation-prone strain is capable of obtaining a selective advantage on cell growth compared with the wild-type counterparts during a short-term adaptive evolution.

### Inactivation of a DNA Repair System Expands the Genotype Space

**FIGURE 3:**
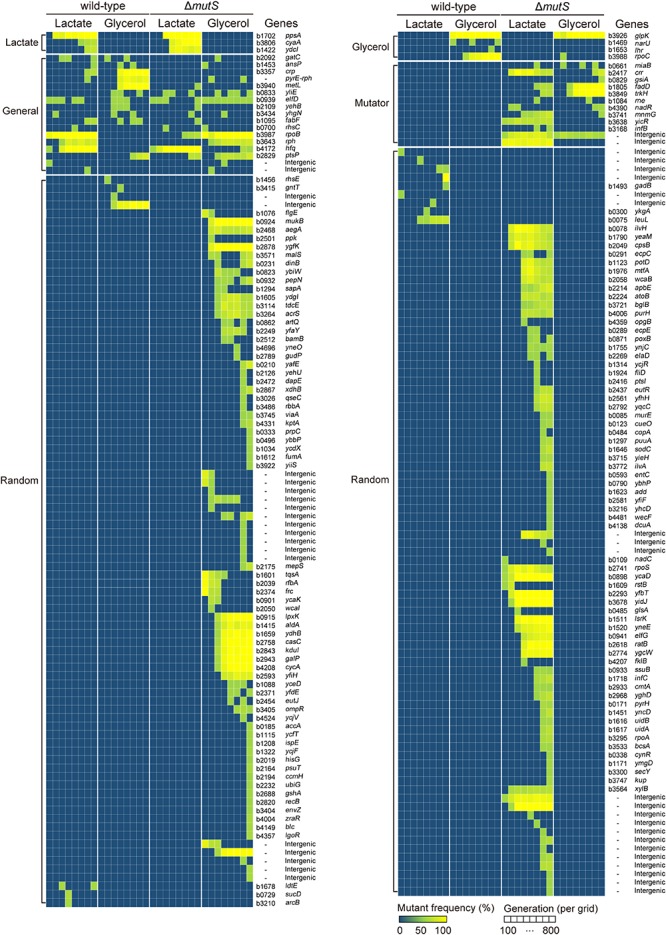
Whole genome resequencing analysis of ALE experiments. Dynamic illustration of the genotype space of the wild-type and the Δ*mutS* lineages in the course of ALE. Each grid on the *X*-axis represents ALE timescale at 100th generation intervals. The *Y*-axis outlines the mutations identified in the corresponding generation intervals which are clustered into subgroups-Lactate (lactate-specific), Glycerol (glycerol-specific), Mutator (mutator-specific) and Random. The mutations were grouped into five different categories – glycerol-specific, lactate-specific, generalist, mutator, and random – in accordance to their recurrence under specific selective pressures. The color gradient in the heat map depicts relative mutation frequency.

To understand the genetic basis of enhanced adaptability in Δ*mutS* mutator populations, we determined the mutation profiles of the ancestral, evolving and end-point populations of the wild-type and the Δ*mutS* mutator lineages. Whole genome re-sequencing was performed for all eight lineages at 100-generation intervals, allowing us to analyze mutation trajectories (Figure 3). As expected, the Δ*mutS* mutator strains accumulated mutations more rapidly than the wild-type strains. The Δ*mutS* mutator strains produced in overall 246 mutations that mapped to 158 genes and 36 intergenic regions, while the wild-type strains harbored 62 mutations mapped to 27 genes and 11 intergenic regions, suggesting that inactivation of *mutS* brings about fourfold increase in mutation rates in bacteria. Such changes in the mutation rate were comparable to, or slightly higher than, the rates reported in other *mutS*-inactivated strains of *Lactobacillus casei* (Overbeck et al., 2017) and *E. coli* C321.ΔA (Wannier et al., 2018). As it was known, frequency of transition mutations were far more pronounced over transversion mutations in all Δ*mutS* mutator lineages (Supplementary Table S3; Modrich, 1991; Tsuru et al., 2015).

We identified a group of genes or intergenic regions recurrently mutated across two or more independent ALE lineages, where selective pressures account for such genetic parallelism in the mutation profiles (Bull et al., 1997; Cooper et al., 2001, 2003; Barrick et al., 2009). The mutated genes were then categorized in accordance to their functional niche – whether the effect of a mutation is confined in homogeneous condition (known as specialist), or potent across a broader range of heterogeneous environments (called generalist) (Kassen, 2002), as summarized in Supplementary Table S3. Examples of generalist mutations in *E. coli* include *rpoB* (RNAP subunit β) and *rph* (truncated RNase PH) which are implicated in translational machinery. In our current design, the sole carbon substrates – lactate or glycerol – supplemented in the minimal media impose selection pressures that favor specialist mutations to arise. So for instance, mutation in glycerol kinase encoding gene *glpK* is known as the specialist mutation in glycerol minimal medium (*Nat Genet, 2006, 38, 1406-1412*; *Nat Commun, 2017, 8:2112*), while mutations in *ppsA* encoding phosphoenolpyruvate synthetase is thought to rewire lactate metabolism in *E. coli* (*Genome Res, 2005, 15, 1365-1372*) thus serving as lactate-specialist mutation. Subsequent analysis revealed that the mutator and the wild-type lineages shared a large proportion of generalist, as well as specialist mutations in a carbon-dependent manner (Supplementary Table S3). Yet, most of the random mutations were found in the mutator lineages (sum of random mutations in all mutators *n* = 167; in all wild-type strains *n* = 16).

The whole-genome re-sequencing demonstrated that the mutations were most prolific in the end-point populations (Figure 3 and Supplementary Table S3). Using the functional analysis using the COG database, we compared the enriched mutations in the end-point populations against the *E. coli* K-12 MG1655 genes. With an assumption that mutation occurs at random throughout the genome, the enrichment levels imply over-representation of relevant functional gene categories or classes. In particular, genes involved in amino acid transport and metabolism (E), secondary metabolites biosynthesis, transport and catabolism (Q) and transcription (K) were more enriched in the mutated genes of end-point populations than the wild-type MG1655 genes, indicating mutation hot spots (Chattopadhyay et al., 2009; Lourenco et al., 2016).

### Short-Term ALE Exerts No Observable Fitness Burden on Mutator Strains

**FIGURE 4:**
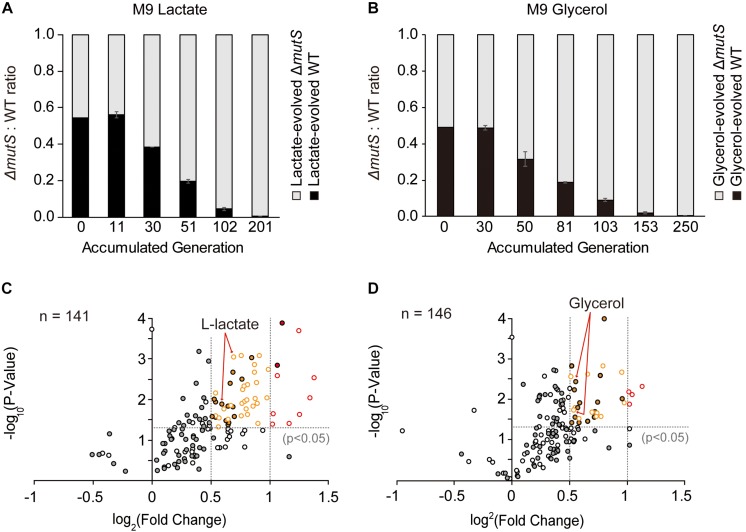
Fitness landscape of the evolved mutator and the wild-type. **(A,B)** Ratio-wise representation of the proportion of the evolved wild-type versus the evolved mutator strains in the course of competition experiment. Each panel depicts competition experiments between **(A)** lactate-evolved mutator and wild-type or **(B)** glycerol-evolved mutator and wild-type. **(C,D)** Relative growth rates of the **(C)** lactate- and **(D)** glycerol-evolved *E. coli* wild-type and Δ*mutS* strains on a panel of nutrient substrates. Volcano plots indicate the logarithmic ratio of growth rates between the lactate- and the glycerol-evolved strains relative to the ancestral strains. Dotted line intersecting the *Y*-axis is the baseline of significance (*p* < 0.05). Substrates with negative growth profile were omitted from the analysis. Filled and blank circles each represent the wild-type and the Δ*mutS* strains. The substrates that lie above the baseline of significance were classified in accordance with the magnitude of fitness improvement in log_2_ scale; those that lie between log_2_ (0.5) and log_2_ (1) (between the dotted lines intersecting the *x*-axis) were shown in yellow, and above log_2_ (1) in red.

To demonstrate whether the Δ*mutS* mutator strain still sustains its competitive edge after undergoing 800 generations of adaptive evolution, we performed a new round of competition experiment involving the end-point lineages of Δ*mutS* and wild-type evolved under lactate or glycerol minimal medium. Two mix-cultures of i. lactate-evolved Δ*mutS* and wild-type and; ii. glycerol-evolved Δ*mutS* and wild-type strains were allowed to competitively evolve in their respective adaptive media. In all instances of competition experiments, the end-point Δ*mutS* strains outcompeted their wild-type competitors by the 201th and 250th generations in lactate- and glycerol-evolved populations respectively (Figures 4A,B). This corroborates similar competition experiment performed in nutritionally rich mouse gut showed that *mutS*-defective *E. coli* pre-adapted in the intestinal environment for 20 days also managed to replace most of its isogenic wild-type competitors in mouse gut (Giraud et al., 2001a).

### Fitness Gain on Alternative Carbon Sources Is More Pronounced in the Mutator Lineages Than the Wild Type

In an effort to relate genotypic changes to phenotypes, we assayed the fitness of the end-point populations across a broad panel of single-carbon environments. As specific growth rate is a commonly proposed representative of bacterial fitness (Nur, 1987), we measured the specific growth rates of both ancestral and end-point populations on 95 different non-adaptive substrates (as described in Methods). It was previously described that the *mutT* hypermutator strain in the long-term evolution experiment and the *mutS* mutator strain that evolved in mouse gut adversely affected fitness landscape on alternative environments presumably due to an accumulation of deleterious mutations (Giraud et al., 2001a; Leiby and Marx, 2014). Yet, fitness assay of *mutL* mutator strain adaptively evolved over 3,000 generations against antibiotics and heavy metals, revealing a stochastic nature of the mutator strain in relation to fitness acquisition or loss in alternative environments (Sprouffske et al., 2018). Phenotype transitions between the ancestral and the evolved strains were summarized in Table 1, where digits in each category refer to the number of relevant substrates in the PM01 microplate. Growth rate data revealed that the number of substrates in Fitness Gain category was similar between the end-point populations of Δ*mutS* mutator strains (*n* = 42 for lactate, *n* = 32 for glycerol), and the wild-type (*n* = 32 for lactate, *n* = 33 for glycerol) (*p* > 0.5, Pearson’s chi-squared test). However, the number of substrates with fitness increase above log_2_ (fold-change) of 0.5 was significantly higher in Δ*mutS* mutator strains (*n* = 37 for lactate, *n* = 16 for glycerol) than in wild-type (*n* = 17 for lactate, *n* = 15 for glycerol) (*p* < 0.0015, Pearson’s chi-squared test). Notably, the number of substrates that fell above log_2_ (fold-change) > 1 was markedly higher in the end-point strains of Δ*mutS* mutator (*n* = 8 for lactate, *n* = 4 for glycerol) than in the wild-type (*n* = 2 for lactate, *n* = 0 for glycerol) (*p* < 0.02, Pearson’s chi-squared test) (Figure 4). Carbon substrates with log_2_ (fold-change) > 0.5, 1.0, *p*-value < 0.05 are listed in Supplementary Table S4.

**TABLE 1:** Tabulation of the Biolog PM01 substrates in accordance to the growth profiles in the eight end-point ALE populations and the corresponding ancestral populations.

	**Glycerol**		**Lactate**	
	**WT**	**Δ*mutS***	**WT**	**Δ*mutS***
Gain of function	8	7	4	4
Loss of function	0	1	1	4
No growth	14	15	19	19
Non-significant	40	39	39	26
Fitness gain (total)	33	32	32	42
Fitness gain (0.5 < log_2_(Fold-Change) < 1.0)	15	16	17	37
Fitness gain (log_2_(Fold-Change) > 1.0)	0	4	2	8
Fitness loss	0	1	0	0

*“Gain of function”: substrates that became accessible (utilizable) by the end-point strains; “Loss of function”: substrates that became inaccessible (non-utilizable) by the end-point strains; “Non-significant”: substrates where changes in growth rates between the ancestral and corresponding end-point strains were non-significant; “Fitness gain”: substrates with significant increase in growth rates in the end-point populations relative to the ancestral strains. They are further classified in accordance to fold-change values in the growth rates; “Fitness loss”: substrates with significant decrease in growth rates in the end-point populations relative to the ancestral strains.*

Apart from the changes in growth kinetics, we observed cases where populations acquired or lost the ability to thrive on alternative carbon or nitrogen substrates. Emergence of such collateral, non-adaptive traits was first realized in the long-term evolution experiments where glucose-adapted strains acquired the ability to metabolize citrate which is a non-utilized carbon source in the wild-type *E. coli* B (Blount et al., 2008). As opposed to the mutators in the long-term evolution experiments, Δ*mutS* mutator strains in this study showed no visible differences in terms of novel gain or loss of function phenotypes following adaptation between the end-point population of the wild-type and the Δ*mutS* mutator lineages (*p* > 0.5 and *p* > 0.1, Pearson’s chi-squared test, for gain and loss of function, respectively). The number of substrates in Gain of Function category was in overall greater than that in Loss of Function. Together, this line of evidence indicated that a short-term ALE does not inflict metabolic capacity of MMR-inactivated mutator strains as seen in the long-term evolution experiments (Leiby and Marx, 2014); rather, this seems to maximize the chance of survival on alternative environments.

### Fitness Analysis of Single Mutations on Alternative Environments

To assess the contributions of individual mutations to fitness on non-adaptive carbon sources, we selected mutation candidates by inferring their roles in carbon and amino acid metabolism (Table 2). Growth rates of the engineered strains harboring a single mutation and their wild-type controls were measured on M9 minimal medium containing adaptive (AC), non-adaptive carbon sources (NC) or both carbon sources (AC + NC). For example, inosine and adenosine which are purine nucleosides are unable to be utilized as sole growth substrates by *E. coli*, instead they can be co-utilized in the presence of other growth substrates such as aspartate (Xi et al., 2000). Therefore, growth rates on inosine and adenosine were measured in M9 minimal medium supplemented with adaptive carbon substrate (AC + NC). Hypoxanthine hydroxylase encoded by *xdhB* constitutes a subdomain of xanthine dehydrogenase and is known to be associated with adenosine degradation pathway in *E. coli* (Xi et al., 2000). The mutation on *xdhB* (860ins861G) from the mutator end-point on glycerol ALE was characterized by a frameshift mutation inflicting the 3′ end of the protein coding region of *xdhB*. The mutation was also identified during the ALE of genome-recoded *E. coli* where selective pressures on amber stop codons may have implicated with read-through of termination codons including that of *xdhB* (Wannier et al., 2018). This evidence suggests the frameshift mutation on *xdhB* is a generalist mutation that impacts fitness via tuning cellular translation efficiency. In our study, *xdhB* mutant (860ins861G) exhibited higher growth rate in the non-adaptive environment (AC + NC), but lower growth rate in adaptive condition (AC), than its wild-type complement (Table 2). On the other hand, gene encoding deoxyadenosine deaminase *add* is involved in nucleotide biosynthesis and metabolism (Remy and Love, 1968). A point mutation in *add* was identified in the mutator end-point on lactate ALE. This mutation (947C > T) is characterized by a substitution of Ala315 to Val positioned toward the C-terminal end of deoxyadenosine deaminase. Single mutant analysis revealed that the mutation conferred fitness advantage on M9 medium containing 0.2% 2′-deoxyadenosine and 0.2% lactate (AC + NC). Yet, the growth rate on 0.2% lactate (AC) was negligible (Table 2). On the other hand, another point mutation on *ilvH* encoding a subunit of acetolactate synthase delivered fitness advantages on all growth media supplemented with 0.2% lactate (AC), 0.2% L-threonine + 0.2% lactate (NC + AC) and another non-adaptive carbon source, 0.2% pyruvate (NC2). The non-adaptive carbon sources are the precursor molecules involved in the biosynthetic pathways of isoleucine and valine which acetolactate synthase is associated with (Guardiola et al., 1974). This mutation (*ilvH* 124A > G) caused substitution of Thr42 to Ala located on the ACT domain with a suggestive role in ligand binding (Grant, 2006). Although the underlying mechanism of how each mutation contributed to fitness increase is elusive, the overall outcome supports the notion that genome-wide accumulation of random mutations provides an impetus for survival on a number of non-adaptive environments (Lynch and Abegg, 2010). Further, such study design may help in understanding the link between genotypes to molecular phenotypes in microbial cells.

**TABLE 2:** The candidate mutations and functionally associated metabolic substrates.

			**Growth rate (±SD)^∗∗^**	
**Gene**	**Mutation^∗^**	**Nutrient supplements**	**SNV**	**Wild-type**
*add*	A316V (947C > T)	Lactate	0.022 ± 0.002	0.021 ± 0.001
		Lactate + 2′-Deoxy Adenosine	0.096 ± 0.003	0.087 ± 0.004
*xdhB*	A287fs (860ins861G)	Glycerol	0.099 ± 0.003	0.108 ± 0.011
		Inosine + Glycerol	0.063 ± 0.004	0.058 ± 0.004
*ilvH*	T42A (124A > G)	Lactate	0.180 ± 0.006	0.143 ± 0.002
		L-trehalose + Lactate	0.207 ± 0.010	0.182 ± 0.007
		Pyruvate	0.141 ± 0.009	0.128 ± 0.004

*^∗^Single nucleotides substitutions are designated by “>”. For example, 947C > T denotes that C is changed to T at nucleotide 947. ^∗^“ins” represents insertion, and “fs” represents frameshift. ^∗∗^SD denotes standard deviation.*

## Discussion

Several studies have shown that in microbial cells, elevated mutation rates can be exploited to facilitate ALE toward better fitness compared with non-mutator cells (Sniegowski et al., 1997; Giraud et al., 2001a; Notley-McRobb et al., 2002; Barrick et al., 2009; LaCroix et al., 2015). Often, MMR-impaired cells have been used as the mutator strain in numbers of ALE applications (Luan et al., 2013; Overbeck et al., 2017; Wannier et al., 2018) because MMR-inactivation were known to generate moderate mutators in *E. coli* (Taddei et al., 1997; Rosche and Foster, 1999; Tenaillon et al., 1999; Loh et al., 2010; Sprouffske et al., 2018). In laboratory settings, the desired ALE results can be achieved when strains become best-adapted for survival in an unfavorable condition. In nature’s perspective, however, evolutionary success also entails the capacity to effectively occupy a range of potential niche. While it is not plausible for a cell to harbor maximum fitness capacity in all circumstances (Law, 1979), microbial cells nonetheless retain the potential to adapt in a new set of conditions. They do this by reserving a small subset of mutator populations that provide a constant influx of random allelic variants (LeClerc et al., 1996; Matic et al., 1997; Rosche and Foster, 1999). This parallels with the idea of stepwise accumulation of intermediary mutations that lead to the emergence of novel phenotypes (Wagner, 2008; Szappanos et al., 2016). In this work, we explored the evolutionary contingencies of mutator ALE by analyzing collateral fitness capacities in our end-point populations across a panel of alternative environments.

*Escherichia coli* MG1655 wild-type and Δ*mutS* mutator strains were adaptively evolved in M9 medium with lactate or glycerol as the sole carbon sources. Fitness levels of the end-point strains increased between 2.2 and 2.7-fold (Figure 1B), which is comparable to fitness increase observed in ALE performed for similar timescales under lactate, glycerol and glucose minimal media (Fong et al., 2005a, b). Generally, it has been known that mutators evolved in short-term give rise to more robust populations compared with their isogenic non-mutator counterparts (LaCroix et al., 2015; Overbeck et al., 2017; Wannier et al., 2018). As opposed to what was expected, there was no appreciable difference in adaptive capacity in terms of fitness gain between the end-point wild-type and Δ*mutS* mutator strain (Figure 1B). This was intriguing given the significant divergence in their mutation profiles (Figure 3 and Supplementary Table S3). The wild-type lineages generated in overall 62 mutations, which is a quarter of what was identified in all four Δ*mutS* mutator strains (246 mutations). In addition, unlike what is considered as usual fitness trajectory of *E. coli* mutators (characterized by a sharp leap in adaptive fitness distinct from that of the non-mutators) (Taddei et al., 1997; LaCroix et al., 2015), the fitness trajectories of the Δ*mutS* mutator strains closely resembled that of the wild-type (Figure 1A). Yet again, the Δ*mutS* mutator strains outcompeted its wild-type counterpart in the competition (Figure 2) despite the growth defect intrinsic in mutators (Ishizawa et al., 2015), demonstrating the selective advantage of mutator populations in adaptation. This observation corroborated the result of similar competition experiments performed in mouse gut (Giraud et al., 2001a) and in glucose chemostat with lag-to-displacement of 60 ± 3.6 generation (Chao and Cox, 1983). The temporal fluctuations in the population (Figure 2A) is likely attributed to “adaption window” characterized by a rapid increase in genetic heterogeneity in a population with varying degree of fitness levels (Barrick et al., 2009; Van Cleve and Weissman, 2015). In such circumstances, mutator populations retain selective advantage for survival in that they can leverage their increased mutation rates, which comes with higher chance acquisition of beneficial mutations, to accelerate adaptation (Sniegowski et al., 1997; Giraud et al., 2001a; Notley-McRobb et al., 2002; Barrick et al., 2009; LaCroix et al., 2015). Together, the results illustrate a strong competitive advantage of mutator strains over wild-type strains in short-term competition.

Variant detection at 100 generation intervals enabled analysis of mutation profile at high resolution. Mutagenic potential of *mutS* inactivation was as high as four-fold than that of basal mutation rate (Supplementary Table S3). Mutation rates of Δ*mutS* mutator strain are roughly comparable with that of *mutT* mutator phenotype in the long-term evolution experiments and Δ*mutS* derivative of recoded *E. coli* strain evolved in short-term, each of which harnessed 62 mutations per 1,000 generations and ∼55 mutations after 1,100 generation respectively (Barrick et al., 2009; Wannier et al., 2018). The mutation rates in the wild-type lineages lied well within the range of those in similar ALE experiments (1 to 40 mutations in ∼ 1000 generations and 2 to 3 mutations in ∼660 generations) (Herring et al., 2006; Barrick et al., 2009; Conrad et al., 2009; Wannier et al., 2018). A group of causative genes reproducibly mutated across independent lineages were classified accordingly (Supplementary Table S3). We first categorized the genes or intergenic regions recurrently mutated in the wild-type and Δ*mutS* mutator lineages, then classified the mutations with regards to their ecological niche width (Supplementary Table S3; Kassen, 2002).

First, the mutations that persist in multiple heterogeneous conditions characterized by a broader niche width is termed “generalist mutations” (Kassen, 2002). The generalist mutations identified in this study were *rpoB* (RNA polymerase subunit β), *hfq* (RNA-binding protein), *rph* (truncated RNase PH), *ptsP* (mannose-specific PTS enzyme IIC component), *crp* (DNA-binding transcriptional dual regulator), and the intergenic region between *pyrE* (orotate phosphoribosyltransferase) and *rph.* The variants of *rpoB and hfq* are highly frequent in the *E. coli* populations adaptively evolved in minimal media (Herring et al., 2006; Conrad et al., 2009; LaCroix et al., 2015), largely owing to their essential role in the transcription (*rpoB*) (Kobayashi et al., 1990) and growth rate dependent effect on bacterial fitness (*hfq*) (Maharjan et al., 2013). Numbers of disabling mutations (frame-shift and non-sense mutations) on *ptsP*, a component of a PTS chain, identified in three independent lineages is consistent with the finding that adaptive evolution of *E. coli* that lacks the PTS system (including deletion of *ptsP*) led to an extensive re-wiring of metabolic flux that contributed to a significant increase in growth rate (Aguilar et al., 2012). Deletion in the intergenic region of *pyrE-rph* is particularly frequent in the adaptive evolution of *E. coli* MG1655 in that the mutation compensates for an intrinsic defect of K-12 strains in pyrimidine biosynthesis (Jensen, 1993; Conrad et al., 2009; LaCroix et al., 2015). These generalist mutations, except for *crp*, were mapped to both the wild-type and the Δ*mutS* mutator lineages (Supplementary Table S3).

Second, the mutations that convey beneficial effect in a confined homogenous environment is called “specialist mutations” (Kassen, 2002). Well-established specialist mutations in lactate ALE include mutations on *ppsA*, *cyaA* and *ydcI* (Conrad et al., 2009). Among them, *ppsA* encodes phosphoenolpyruvate synthetase which is associated with conversion of pyruvate into phosphoenolpyruvate or vice versa. Adenylate cyclase encoding gene *cyaA* is associated with cAMP production and is implicated in the utilization of non-PTS carbon sources. For this reason, *cyaA* mutants are frequently seen in ALE experiments in non-PTS carbons such as lactate and glycerol (Herring et al., 2006; Conrad et al., 2009; Peabody et al., 2017). Moreover, *ydcI* is a gene that encode putative DNA-binding transcriptional repressor that is thought to be implicated in cellular stress response (Solomon et al., 2014). The recurrence of *ydcI* mutants in other ALEs on minimal and rich media (Conrad et al., 2009; Liu et al., 2017) also suggests their role in globally mediated regulation in fitness improvement through some unknown mechanisms. The variants of all three genes were identified across lactate-evolved wild-type and the Δ*mutS* mutator lineages (Supplementary Table S3). As for the glycerol specialist mutations, previous studies demonstrated causal link of *glpK* (glycerol kinase) and *rpoC* (β’ subunit of RNA polymerase) mutation in growth rate improvements in glycerol ALE (Conrad et al., 2011; Sandberg et al., 2017). As an essential component of RNA polymerase, *rpoC* is thought to be selected in virtually any sub-optimally fit bacterial population (Murphy and Cashel, 2003). The variants of *glpK* and *rpoC* were also mapped across glycerol-evolved wild-type and the Δ*mutS* mutator lineages (Supplementary Table S3).

Third, there were only few causative variants identified under wild-type specific (*crp*) and Δ*mutS*-specific (*trkH*) categories. The variant of *crp* was reported to contribute to fitness increase in *E. coli* strains adaptively evolved in non-preferential carbon sources including lactose (Quan et al., 2012). K^+^ transporter protein encoded from *trkH* is involved in ion efflux regulation. It was reported that a non-synonymous mutation on *trkH* arose from an antibiotic challenge resulted in mild resistance against non-target antibiotics (Lazar et al., 2013). Emergence of an off-target, collateral effect against antibiotics in ALE experiments was also documented in mutator populations adaptively evolved under nutrient limitations for 3000 generations (Sprouffske et al., 2018).

Lastly, in addition to the generalist and specialist mutations, the remaining 183 mutations were found to be mutually exclusive and mapped uniquely to one of the eight ALE lineages (Supplementary Table S3). These mutations were designated as random mutations. Notably, nearly all of the random mutations were found in the *mutS*-inactivated lineages (*n* = 167), where lactate- and glycerol-adapted Δ*mutS* mutator lineages harbored 83 and 84 of random mutations, while the corresponding wild-type lineages retained only 4 and 12 random variants respectively. Interestingly, genes that have not been registered in the ALE mutation metadatabase (ALEdb) were newly identified in the mutator lineages (Phaneuf et al., 2019). It is also worth noting that, whereas the profiles of the generalist and the specialist mutations were largely consistent across all eight ALE lineages, distribution of the random mutations was highly biased toward the mutator lineages.

Genetic load incurred by a gradual accumulation of deleterious mutations is often presented as an evolutionary tradeoff in mutator populations in exchange for their accelerated pace of evolution. Mutators inflicted with genetic burden are either outcompeted by fitter sub-lineages (Giraud et al., 2001a), or rescued with the emergence of anti-mutator genotypes that alleviate the genetic burden (Wielgoss et al., 2013). The fact that the evolved mutator strains managed to outcompete the isogenic wild-type controls indicates that short-term evolution in minimal medium imposed no observable genetic burden to the mutator strains (Figures 4A,B). This seems to indicate that the cost of mutations accumulated in the mutator lineages through a short-term evolution did not outweigh the benefit (Giraud et al., 2001a).

With evidence suggesting that a successive accumulation of random mutations may, in turn, leads to secondary adaptive mutation (Wagner, 2008; Lynch and Abegg, 2010; Szappanos et al., 2016), growth rate measurement on a number of non-adaptive carbons was performed to prove whether these lines of genotypic changes translates to phenotypic changes. Previous studies on the populations in the long-term evolution experiments observed an extensive antagonistic pleiotropy of the 20,000 and 50,000 generation populations on a range of non-specialized environments, where the performance of mutator strains on non-adaptive environments was worse off than wild-type strains (Leiby and Marx, 2014). It is speculated that global metabolic erosion observed in the mutator lineage results from the gradual accumulation of mild-to-moderately deleterious non-synonymous mutations imposing a severe fitness burden (DePristo et al., 2005; Wielgoss et al., 2013). In this study, however, it was observed that the mutators adaptively evolved in short-term performed better across a spectrum of alternative nutrient substrates than the wild-type strains. It is possible that the sheer abundance of beneficial generalist mutations that affect global regulatory network drew the difference in the fitness profile. Nonetheless, single mutation analysis of *add* (947C > T), *xdhB* (860ins861G) and *ilvH* (124A > G) identified in the end-point populations of Δ*mutS* mutator lineages demonstrated fitness advantage on respective non-adaptive nutrient substrates (Figure 4). Together, this evidence seems to suggest that random expansion of genotype space driven by short-term laboratory evolution of mutators facilitates adaptation across a broader spectrum of fitness landscapes.

Overall, a moderate increase in mutation rates confers evolutionary edge in survival across a span of heterogeneous environments. In addition, short-term mutator ALE may serve as a tool of choice in generating versatile microbes with a strong survival advantage in fluctuating environments without observable genetic load. Use of high-throughput genotype and phenotype screening techniques enabled identification of random mutations likely causative in alternative environments.

## Data Availability

Whole genome resequencing data generated during the current study are available in the EMBL Nucleotide Sequence Database (ENA) with primary accession number PRJEB32586.

## Author Contributions

B-KC conceived and supervised the study. B-KC, MK, and KK designed the study. MK and KK performed the experiments, analyzed the data, and wrote the manuscript with contributions from B-KC, DC, SC, SK, and BP.

## Conflict of Interest Statement

The authors declare that this study received funding from the Novo Nordisk Foundation. The funder was not involved in the study design, collection, analysis, and interpretation of data, the writing of this article or the decision to submit it for publication.
